# Does economic empowerment protect women from intimate partner violence?

**DOI:** 10.5249/jivr.v3i1.76

**Published:** 2011-01

**Authors:** Koustuv Dalal

**Affiliations:** ^a^Department of Medical and Health Sciences, Centre for Medical Technology assessment & Division of Social Medicine and Public Health Sciences, Linkoping University, Sweden.

## Abstract

**Background:**

The current study compared working and non-working groups of women in relation to intimate partner violence. The paper aims to explore the relationship between women's economic empowerment, their exposures to IPV and their help seeking behavior using a nationally representative sample in India.

**Methods:**

This was a cross sectional study of 124,385 ever married women of reproductive age from all 29 member states in India. Chi-square tests were used to examine differences in proportions of dependent variables (exposure to IPV) and independent variables. Multivariate logistic regressions were used to assess the independent contribution of the variables of economic empowerment in predicting exposure to IPV.

**Results:**

Out of 124,385 women, 69432 (56%) were eligible for this study. Among those that were eligible 35% were working. In general, prevalence of IPV (ever) among women in India were: emotional violence 14%, less severe physical violence 31%, severe physical violence 10% and sexual violence 8%. For working women, the IPV prevalence was: emotional violence 18%, less severe physical violence 37%, severe physical violence 14% and sexual violence 10%; whilst for non-working women the rate was 12, 27, 8 and 8 percents, respectively. Working women seek more help from different sources.

**Conclusions:**

Economic empowerment is not the sole protective factor. Economic empowerment, together with higher education and modified cultural norms against women, may protect women from IPV.

## Introduction

Intimate partner violence (IPV) against women is now a well recognized public health and human rights problem associated with different health, family, social and economic effects.^1,2,3^IPV, in all forms, occur every day in all parts of the world cutting across age, religions, societies, ethnicities and geographical borders.^4^To better understand the extent and nature of the problem of IPV, numerous studies have been conducted in industrialized countries.^5,6^However, considering diverse culture and social contexts developing countries demand much more context-dependent studies on IPV as its risks and effects are relatively unknown in these countries.^7,8^

Social  science  theories  of  IPV  have  explained  a  wide range of causes of IPV, such as men’s pathology (abnormal personality traits/alcoholism), power relation, cultural norms and institutional practices, learned behavior theory.^7,9,10,11^However, no single theory has sufficient empirical support to cover the entire phenomena.^7,13,14^Some experts resort to economic issues to explain violence against women by their husbands or intimate partners. One group propounds that women employment generates more economic resources for them which results in a decrease in violence; while other groups advocate that violence will increase as husbands/ intimate partners attempt to compensate for enhanced women status and independence due to employment.^15,16,17,18^

The United Nations strongly recommended economic empowerment of women as a protective factor for violence against women in its Beijing declaration.^19^To argue these advocacies, studies from industrialized countries have focused on the working status and economic empowerment of women as protective factors and have called for more studies especially from developing countries.^4,6,17,20,21,22,23^

The current study compared non-working and working groups of women for their exposure to IPV. The study aims to explore the relationships between women’s economic empowerment, their exposures to IPV and their help seeking behavior, using a nationally representative sample in India.

## Methods

The Indian government has initiated the National Family Health Surveys (NFHS) to provide reliable quality data on population and health indicators. After NFHS-1 (1992-93) and NFHS – 2 (1998 -99), the government of India has recently completed the NFHS -3 (2005 -06). Besides other important demographic and health indicators, NFHS–3 has highlighted economic indicators and domestic violence against women. A record number of 124,385 women of reproductive age (15–49 years) were interviewed from 29 member states of India.

**NFHS -3 sampling and data collection**

NFHS-3 was funded by the United Sates Agency for International Development (USAID), the United Kingdom Department for International Development (DFID), the Bill and Melinda Gates Foundation, UNICEF, UNFPA, and the Government of India (GOI). Technical assistance was provided by Macro International, Maryland, USA, which is well experienced in conducting Demographic and Health Surveys (DHS) in developing countries.  Eighteen research organizations (including five Population Research Centres established  by  the  GOI  in  various states)   conducted   the fieldwork for NFHS-3.

Fieldwork was conducted in  two  phases  from  November 2005 to August 2006. In the first phase, fieldwork was conducted in 12 states: Andhra Pradesh, Assam, Chhattisgarh, Delhi, Gujarat, Maharashtra, Meghalaya, Orissa, Punjab, Rajasthan, Uttar Pradesh, and West Bengal; whilst in the second-phase, the study was conducted in the remaining 17 states: Arunachal Pradesh, Bihar, Goa, Haryana, Himachal Pradesh, Jammu and Kashmir, Jharkhand, Karnataka, Kerala, Manipur, Madhya Pradesh, Mizoram, Nagaland, Sikkim, Tamil Nadu, Tripura, and Uttaranchal.

The NFHS -3 was targeted to focus on a large number of key indicators of ever married women in the reproductive age group of 15-49 years. Hence, this paper used interview data from ever-married women in their reproductive age from the NFHS -3.

The NFHS -3 used the 2001 population census to determine the sample size from each area.

The initial targeted sample size (for completed interviews) was 4,000 ever-married women in states with a  population of more than 30 million, 3,000 ever-married women in states with a  population between 5 and 30 million, and 1,500 ever-married women in states with a population of less than 5 million.

NFHS -3 followed a uniform sample design procedure using probability proportional to population size (PPS). The rural sample selections were made in two stages: in the first stage selection of Primary Sampling Units (PSUs), which were villages, with PPS and in the second stage, random selection of households within each PSU. The urban sample selections were made in three stages: in the first stage selection of PSUs, which were municipality wards, with PPS. In the next stage, from each sample ward one census enumeration block (CEB) was randomly selected. In the final stage, there was random selection of households within each selected CEB.

With more than 96% response rate, a total of 109,041 households were interviewed. And with more than 90% response rate, from the selected households 124,385 eligible women completed face-to-face interviews. However a more detailed description of the sampling procedure is reported in the NFHS –3 final reports 2007.^24^

**Questionnaire**

The NFHS -3 questionnaires provide detailed data on women’s background, reproductive history, utility of family planning methods, fertility preferences, antenatal and delivery care, child care and nutrition, child mortality, adult mortality, awareness of and precaution against sexually transmitted diseases, marriage and sexual behavior, empowerment and social indicators and domestic violence. Primary interests for this  paper  were  the  domestic  violence  module and socioeconomic variables.

**Dependent variable**

Domestic violence or intimate partner violence was defined as exposure to one or several of the following experiences:

a)emotional violence: husband ever i) humiliated her, ii) threatened her with harm, iii) insulted or made feel bad.

b)less severe physical violence: husband ever i) pushed, shook or threw something, ii) slapped, iii) punched with fist or something harmful, iv) kicked or dragged.

c)severe physical violence: husband ever i) tried to strangle or burn, ii) threatened or attacked with knife/gun or other weapons.

d)sexual violence: husband ever i) physically forced sex when not wanted, ii) forced other sexual acts when not wanted.

**Independent variables**

 Age, rural/urban dwelling, educational achievement, and religion were included in demographic characteristics. The age variable was classified into seven age groups (15 – 19, 20 – 24, 25 – 29, 30 – 34, 35 – 39, 40 – 44 and 45 – 49). Education was classified into four groups: no education, primary education, secondary and higher education. Religion in the NFHS – 3 consisted of Hindu, Muslim, Christian, Sikh, Budhist/ Neo-Budhist, Jain, Jewish, Parsi/Zoroastrian, no religion, Donyi polo and others. However, during the analysis it was found that apart from the first three religions, respondents from other religions reported very low rates of IPV. Those religions were then merged and formed the new heading others.

India is well known for its caste system. In the current study, four categories of castes were used: scheduled castes (SC), scheduled tribes (ST), other backward classes (OBC) and others who are not within these three groups. People belonging to these castes are protected by the constitution of India. They are entitled to receive extra facilities by means of positive discrimination in educational, employment and other developmental opportunities for their socioeconomic empowerment.^24^

In the previous national health survey (NFHS-2) some new variables were introduced to measure the housing facilities such as kachha (household materials are mud and clay), semipucca (household materials are a mixture of cement and mud) and Pucca (cemented). In the current study housing facilities were used as a demographic factor.^24^

**Socioeconomic  characteristics   of the   respondent’s   family** included wealth index, sex of household head, family’s health insurance coverage and family coverage under Indian governments below poverty line (BPL) protection facility and respondents’ bank account.

Wealth index is a widely used measurement of economic status used to ascertain the equity of health programs in publicly or privately provided services. The main objectives of wealth index are to measure ability to pay for health services and the distribution of services among the poor. Wealth index was validated and used in several demographic and health surveys in different countries. The Wealth Index is a composite measure of the cumulative living standard of a household. It is calculated by using data on a household’s ownership of selected assets, e.g. radio, televisions and bicycles; materials used for construction of house, types of water-access and use of sanitation facilities. Wealth Index uses a generated statistical procedure known as the principal components analysis and places individual households on a continuous scale of relative wealth. The scale is standardized in relation to a standard normal distribution with a mean of zero and a standard deviation of one. These standardized scores are then used to create the groups that define wealth quintiles as: Poorest, poorer, middle, richer and richest. The wealth index used in India was introduced by Rutstein and Johnson (2004) and includes any item that may reflect economic status, specifically most household assets and utility services, including country-specific items.^25^

In patriarchal societies like India, sex of the household head is important as it often leads to risk factors for domestic violence against women.^7,23^Therefore the current study also considered sex of household head.

As per the Government of India, the poverty line for urban areas is 296 INR (Indian Rupees) per month and for rural areas 276 INR per month, i.e. people in India who earn less than 10 INR per day live below the poverty line. As per 2007 statistics, nearly 27 percent of rural Indians and approximately 11 percent of urban Indians live below the poverty line (BPL).^26^Families living below the poverty line receive subsidized food from the government through public distribution systems (PDS).

**Economic empowerment indicators** assessed included respondents working status, working facilities, employment status, and income comparison with partners and exposure to bank accounts.

Working status was assessed by whether respondent was working or not. Working facilities were measured by respondent’s work at her own home or away from home. Employment status had three alternatives, whether respondent worked all year round, seasonal or occasional. Income comparison (earnings) of the respondent was classified into four groups: respondent earns more than partner, less than partner, earns same as partner and partner had no income.

**Help seeking behavior**

Finally the study considered the help seeking behavior of IPV victims. Options for seeking help for IPV were: Sought help from: own family members, husband’s family members, current/former boyfriend, social service organization, friend, police, religious leader, physician or someone else. The analysis found that less than 0.2% of IPV victims sought help from current/former boyfriend, social service organization, friend, religious leader and physician.  Hence, in the analysis only four sources of help (own family members, husband’s family members, police and someone else) were considered.

**Statistical analysis**

Chisquare test was used to examine differences in proportions of exposure to IPV of the working and non-working women by demographic and socioeconomic variables; and of the help seeking behavior by working status and IPV exposures. For example, for working women the variables of interest were selected first and the crosstabulation was then run with chisquare tests for dependent and demographic variables presented in Table 1. The same was performed separately for non-working women. Therefore, for each dependent variable (say, emotional violence) there are different figures for working and non-working women according to demographic variables. For easy comparison, those figures were presented in the same table with respective p-values, generated by chisquare tests (Table 1andTable 2). Multivariate logistic regressions were run with all the variables of economic empowerment of women (working or not, work at home or away from home, employment status and earning comparison with husband)  to assess the independent contribution of these variables in predicting exposure to IPV. Due to the large number of observations, the analysis considered 99% confidence intervals in the logistic regression analysis. PASW 18 was used for data analysis.

**Ethical permission**

The standards for ethical and safety recommendations for research on domestic violence set by the World Health Organization (WHO) were strictly followed for data collection of the domestic violence module.^27^It aimed to ensure women’s safety and maximum disclosure of actual violence. NFHS - 3 offered adequate training and support to fieldworkers, secured informed consent and guaranteed privacy of the respondents. The study received ethical permission from the Institutional Review Board, ORC Macro International, USA.

## Results

Out of 124,385, 69432 (56%) women had responded to the IPV related questions. In total 43669 (35%) women were working. In general, prevalence of IPV (ever) among women respondents was: emotional violence 14%, less severe physical violence 31%, severe physical violence 10% and sexual violence 8%. However, for working women the IPV prevalence was: emotional violence 18%, less severe physical violence 37%, severe physical violence 14% and sexual violence 10%; while for the non-working women the rate was 12, 27, 8 < 8 percents, respectively.

**Demographics**

For both working and non-working women, proportions of exposure to less severe physical violence had a slightly elevated trend and that of sexual violence had a slightly relegated trend with increase in their age. Urban women were more exposed to emotional and less severe physical violence while rural women were more exposed to sexual violence. Education had the usual relationship, i.e. higher education provided less exposure to IPV. Almost all demographic characteristics of the respondents demonstrated heightened proportions of IPV exposures for the working women compared to non-working women. However, for women with higher education, the proportions of IPV exposures were by and large the same amongst working and non-working women. In general, women of Christian religion, general (uncategorized) castes and of pucca (cemented/concrete) housing facilities demonstrated lowest  exposure to IPV(Table 1).

**Table 1:Comparison of violence exposure between non-working and working women according to demographics:** 

Variables	Non-working Women (NNW)	Emotional violence		Less severe physical violence		Severe physical violence		Sexual violence		Working Women(NW)
		% of NNW	% of NW	% of NNW	% of NW	% of NNW	% of NW	% of NNW	% of NW	
Age		p=0.519	p=0.117	p=0.000	p=0.002	p=0.031	p=0.109	p=0.000	p=0.004	
15-19 years	2346	11	16	23	30	7	11	10	12	679
20-24years	8054	12	17	27	36	7	13	9	11	2657
25-29years	9863	12	18	28	38	9	13	8	10	5082
30-34years	8587	12	17	28	37	8	13	8	9	5785
35-39years	6620	13	19	28	38	8	15	8	10	5186
40-44years	4723	12	19	26	36	8	14	6	9	3742
45-49years	3544	12	18	27	36	8	14	6	8	2445
Residential area		p=0.000	p=0.069	p=0.000	p=0.000	p=0.000	p=0.001	p=0.000	p=0.001	
Urban	21677	11	17	25	35	6	13	6	9	8776
Rural	22059	13	18	30	38	10	14	10	10	16800
Education		p=0.000	p=0.000	p=0.000	p=0.000	p=0.000	p=0.000	p=0.000	p=0.000	
No education	14689	16	21	39	44	13	17	11	11	12805
Primary	6720	15	21	33	40	10	17	9	11	3999
Secondary	18516	9	15	20	29	5	9	5	8	6559
Higher	3808	5	7	8	11	1	2	2	3	2209
Religion		p=0.000	p=0.000	p=0.000	p=0.000	p=0.000	p=0.000	p=0.000	p=0.000	
Hindu	31885	12	18	27	38	8	14	8	10	19641
Muslim	6556	14	20	24	40	9	15	10	13	2024
Christian	2989	11	14	18	24	6	8	3	6	2709
Others	2306	10	19	24	39	7	13	5	9	1201
Caste		p=0.000	p=0.000	p=0.000	p=0.000	p=0.000	p=0.000	p=0.000	p=0.000	
Scheduled caste	7154	15	22	37	47	12	19	10	12	4786
Schedules tribe	4336	14	18	25	35	7	13	7	9	4783
Other backward class	13576	13	18	31	38	9	14	8	8	8524
Others	16793	10	14	22	30	5	10	7	9	6443
Housing facility		p=0.000	p=0.000	p=0.000	p=0.000	p=0.000	p=0.000	p=0.000	p=0.000	
Kachha	3836	17	21	39	45	13	17	13	13	3482
Semi-Pucca	14014	15	19	34	41	11	15	10	11	11225
Pucca	23850	10	15	22	30	6	11	5	7	10255

P-value of chi-square test.

**Family level socioeconomic indicators**

Higher economic solvency of women’s families resulted in lower proportions of IPV exposure. Female headed households had more IPV exposure than male headed families. Interestingly, working women from female headed families had 1.5 to 2 times more IPV exposures than from male headed families. Families with health insurance coverage had nearly half IPV exposure rate than that of the families without insurance coverage. Poverty did not provide a high difference in the rate of  PV  exposures  among  families  with and without BPL card(Table 2).

**Table 2:Comparison of violence exposure between non-working and working women according to family level characteristics:** 

Variables	General Women (NNW)	Emotional violence		Less severe physical violence		Severe physical violence		Sexual violence		Working Women(NW)
		% of NNW	% of NW	% of NNW	% of NW	% of NNW	% of NW	% of NNW	% of NW	
Wealth index		p=0.000	p=0.000	p=0.000	p=0.000	p=0.000	p=0.000	p=0.000	p=0.000	
Poorest	4876	19	23	43	47	15	19	14	13	4844
Poorer	5824	17	21	39	44	13	17	13	11	5269
Middle	7765	14	18	33	38	11	15	10	10	5757
Richer	10912	11	17	27	35	7	12	6	9	5091
Richest	14359	7	11	14	18	3	4	4	4	4613
Sex of household head		p=0.037	p=0.000	p=0.426	p=0.000	p=0.001	p=0.000	p=0.052	p=0.000	
Female	4656	13	23	27	40	9	19	8	13	3780
Male	39080	12	17	27	36	8	13	8	9	21794
Covered by a health insurance		p=0.000	p=0.000	p=0.000	p=0.000	p=0.000	p=0.000	p=0.000	p=0.000	
No	37980	12	18	29	38	8	14	8	10	23309
Yes	2838	8	10	17	19	3	6	5	6	1258
Covered by BPL card		p=0.000	p=0.044	p=0.000	p=0.000	p=0.000	p=0.000	p=0.000	p=0.07	
No	33703	11	18	26	36	7	13	7	9	17994
Yes	8018	14	19	33	40	11	16	10	10	6991

P-value of chi-square test.

**Individual economic indicators**

As indicated by the odds ratios (OR) working women were more likely to be abused by their husbands than their non-working peers. Women who traveled away from home for work were more likely to be emotionally and physically (severe) abused and less likely to be sexually abused by their husbands compared to their peers who worked at home. Seasonal and occasional working women were more likely to be physically (severe) abused than the women who worked regularly all year rounder. Similarly, seasonal working women were more likely to be sexually abused than women in regular employment.  Women who earned less than or the same as their partners experienced less violence compared to their peers who earned more than their partners. However, husbands with no income were more likely to abuse their wives compared to the husbands who earned less than their wives. In contrast, women with bank transactions were less likely to be victims of IPV compared to their peers who had no bank transactions (Table 3).

**Table 3:Exposure to IPV according to variables of economic empowerment:** 

Variable	(n)	Emotional violence		Less severe physical violence		Severe physical violence		Sexual violence	
		% of n	OR ( 99% CI)	% of n	OR ( 99% CI)	% of n	OR ( 99% CI)	% of n	OR ( 99% CI)
Working status									
Not-working	(43736)	12	1.0	27	1.0	8	1.0	8	1.0
Working	(25574 )	18	1.60(1.53 – 1.68)***	37	1.55 (1.40 – 1.60)***	14	1.81(1.72 – 1.90)***	10	1.26(1.16 – 1.33)***
Works at home or away									
At home	(6368)	16	1.0	36	1.0	12	1.0	11	1.0
Away	(23141)	19	1.19 (1.10 – 1.28)***	38	1.08 (1.02 – 1.14)*	14	1.22 (1.12 – 1.33)***	10	0.90 (0.82 – 0.98)*
Employment status									
All round the year	(18913)	18		35	1.0	13		9	1.0
Seasonal	(9344)	19		41	1.29 (1.28 –1.35)***	14		11	1.25 (1.13 –1.39)***
Occasional	(1254)	19		40	1.20 (1.07 – 1.35)**	13		11	
Earns more than partner									
More than	(2016)	21	1.0	40	1.0	13	1.0	9	1.0
Less than	(13231)	17	0.73 (0.65 – 0.83)***	38	0.90 (0.82– 0.99)*	13	0.70 (0.62– 0.80)***	10	
Same	(2099)	16	0.68 (0.58 – 0.80)***	33	0.74 (0.65– 0.85)***	11	0.58(0.48– 0.70)***	7	0.31(0.58 – 0.91)**
Partner no income	(425)	28	1.42 (1.12 – 1.80)***	47	1.32 (1.10 – 1.64)**	22	1.38 (1.07 – 1.78)*	12	1.40 (1.10 – 1.94)*
Respondent has bank account									
No	(56223)	15	1.0	33	1.0	11	1.0	9	1.0
Yes	(13106)	11	0.67 (0.62 – 0.72)***	21	0.54 (51 – 58)***	7	0.58 (0.53 – 0.64)***	6	0.61(0.55 – 0.68)***

*** P<0.001, **P<0.010, *P<0.5

Proportionally, working women sought more help than their non-working peers.  However, it was also observed that they sought higher proportions of help from someone else and from their own family members compared to husbands’ family for any IPV. Least help were sought from the police (Table 4).

**Table 4:Help-seeking sources according to working status and exposures to IPV:** 

	Sought help from own family members	Sought help from husband’s family	Sought help from someone else	Sought help from police
Working status				
Non-working women Ψ	16%	6%	22%	0.5%
Working women Ψ	19%	8%	27%	1%
Violence exposure				
Emotional Ψ	28%	11%	61%	2%
Less-severe physical Ψ	18%	8%	74%	1%
Severe physical Ψ	31%	14%	56%	2%
Sexual Ψ	22%	10%	69%	2%

Ψ chi-square test:  p-value< 0.001

## Discussion

The current study found that the working status of women in India is not a protective factor for violence against women. It was a unique study with national representative data from all the 29 member states of India and demonstrated working women’s elevated proportions (in chi-square test) of IPV exposures for all the demographic and family level variables, compared to their non-working peers. Odds ratios with statistical significances also demonstrated the elevated IPV exposures for working women. Economic empowerment by means of earning is not the only protective factor for IPV, at least in the Indian context. However working women sought more help than non-working women.

Demographic factors for IPV exposures found in the current study were in line with previous findings from developing countries.^3,6,7,16,23,28,29^Family level socioeconomic factors demonstrated some interesting relationships. Proportions of exposure to IPV of non-working women were the same or close to equal in female and male headed families. However, working women in female headed families were more exposed to IPV than male headed families. The reasons might be the Indian patriarchal system where throughout one’s lifetime, a woman is dependent on men such as her father, husband and son due to economic and social customs and where working women are viewed as inferior.^30^Indian women culturally still believe that men are for income earning and women are for household work. However if household work, care work and other voluntary work are treated as a source of income and included into the national income account then probably the  notion will gradually change. The same ideology has also been advocated by the Commission on Social Determinants of Health of the World Health Organization.^31^

Families who have health insurance coverage generally have higher social roles, especially in a country like India where more than a quarter of the entire population lives below the poverty line. Furthermore, the insured households are  more  health  concerned – which might  be  a  reason  to produce lower IPV exposure.^31^

Considering wealth index, the poorest women were most likely to experience domestic violence than the richest women. This finding confirms the WHO findings that poverty disproportionately influences violence against women.^6^ However, in the poorest strata, IPV exposures were not different since the BPL card did not demonstrate any significant difference.

Proportion of exposure to severe physical violence for working women was more than double in urban India than non-working women. The probable reason might lie with the urban husband’s elevated attitude of physically hurting his wife due to his superiority complex.^32^Historically, according to societal norms in India, the husband was the bread-winner of the family and women worked only in the household.^2,7,30^Women now work for economic benefit and this might go against the long-nurtured societal beliefs of the husband and the notion of a husband’s empowerment in the family, thereby inducing domestic violence against women. As the wives who earn more than their husband are more likely to be abused, the actual reasons for IPV victimization of women in India might be explained through complex phenomena including socioeconomic inequality in power and rights, familial hierarchy, and marriage related norms.^31,1,28,29^

It has been established a long time ago that education is a protective factor for IPV.^2,6,7,23,28^The current study demonstrated that economic empowerment, along with higher education was an effective protection for IPV as the exposure rates were low enough (2 – 11%) compared to secondary (5 -29%) or lower/no educated women (11 – 44%). However compared to non working peers, working women with higher education had greater IPV exposures. Probable reason might be the ego factors of the husbands and gender biases in the Indian society.^29,30,32^The reasoning was also supported by the fact that women who earned more than their husbands and who worked away from home were more likely to be abused (Table 3).

The current study had an important advantage when compared with other surveys.  It was nationally representative allowing for conclusions to cover the entire nation. However, weaknesses of the study (under NFHS -3) deserve some acknowledgement. NFHS – 3 used the same technical facilities as different demographic and health surveys (DHS) from Macro International, USA.  Research based on data from Nicaragua, Kenya and Colombia suggested that DHS surveys still under-estimated the extent of IPV when compared with other surveys like the WHO’s multi-country survey on gender-based violence and other specialized violence surveys.^8^Thus, the frequencies reported here might represent an underestimation. The cross-sectional design of this study did not allow for causal inference which warranted longitudinal study designs to firmly establish causal links. Qualitative study of working and non-working women related to IPV exposures are highly warranted for better insight into the field to specifically identify the power relationships, gender inequality and social norms. At the same time, studies on partner’s characteristics and influence of other women for perpetuating domestic violence were also warranted. Logistic regression considered only the variables of economic empowerment. Potential confounding effects of demographics and family level variables were not measured here. Several previous studies from India had identified the possible confounding effects of education, religion and rural-urban residency on IPV exposure.^2,5,24,29,30,32^However, the current study measured the effects of economic empowerment alone on IPV exposure.

The recent report of the Commission on Social Determinants Of  Health   has   strongly  recommended  addressing  gender biases in the structures of society, for developing and financing policies targeting to close the gap in education and skills to better support women’s economic participation.^31^The current study extends strong support to those recommendations, and at the same time also adds the demand to consider the culture of the societies where at least female headed families should learn to provide more protection against wife abuse, i.e. cultural norms to empower women. Economic empowerment of women could not work alone to protect from IPV. However, the current study demonstrates that working women have sought more help for IPV related issues which means that more economic empowerment, along with higher education, may provide women with the awareness, ground, protesting platform and eventually the protective factors against IPV.
